# ‘Who does this patient belong to?’ boundary work and the re/making of (NSTEMI) heart attack patients

**DOI:** 10.1111/1467-9566.12778

**Published:** 2018-06-28

**Authors:** Helen Cramer, Jacki Hughes, Rachel Johnson, Maggie Evans, Christi Deaton, Adam Timmis, Harry Hemingway, Gene Feder, Katie Featherstone

**Affiliations:** ^1^ Centre for Academic Primary Care, Population Health Sciences, Bristol Medical School University of Bristol Bristol UK; ^2^ Centre for Trials Research Cardiff University Cardiff UK; ^3^ Department of Public Health and Primary Care, School of Clinical Medicine University of Cambridge Cambridge UK; ^4^ Department of Cardiology Barts and The London NHS Trust London UK; ^5^ UCL Partners Farr Institute of Health Informatics Research London UK; ^6^ School of Healthcare Sciences Cardiff University Cardiff UK

**Keywords:** emergency care, heart disease, hospitals, ethnography, quality of care

## Abstract

This ethnography within ten English and Welsh hospitals explores the significance of boundary work and the impacts of this work on the quality of care experienced by heart attack patients who have suspected non‐ST segment elevation myocardial infarction (NSTEMI) /non‐ST elevation acute coronary syndrome. Beginning with the initial identification and prioritisation of patients, boundary work informed negotiations over responsibility for patients, their transfer and admission to different wards, and their access to specific domains in order to receive diagnostic tests and treatment. In order to navigate boundaries successfully and for their clinical needs to be more easily recognised by staff, a patient needed to become a stable boundary object. Ongoing uncertainty in fixing their clinical classification, was a key reason why many NSTEMI patients faltered as boundary objects. Viewing NSTEMI patients as boundary objects helps to articulate the critical and ongoing process of classification and categorisation in the creation and maintenance of boundary objects. We show the essential, but hidden, role of boundary actors in making and re‐making patients into boundary objects. Physical location was critical and the parallel processes of exclusion and restriction of boundary object status can lead to marginalisation of some patients and inequalities of care (A virtual abstract of this paper can be viewed at: https://www.youtube.com/channel/UC_979cmCmR9rLrKuD7z0ycA).

## Introduction

The identification and treatment of heart attacks represent a significant point of alignment and tension in which different professions must work closely together within specific locations, relatively short timeframes, and with high stake outcomes for patients. Some types of heart attack are more difficult to identify, manage and treat than others and uncertainty in diagnosis makes the necessary focus and coordination of tasks, people and patients particularly challenging. Understanding processes underlying the work of diagnosis, care delivery and coordination is particularly important because of variation between hospitals in survival after admission for a heart attack. Within this paper we articulate these processes through the lens of boundaries and boundary objects. We start by outlining the key clinical aspects of NSTEMI care and the wide variation in cardiac patient survival rates following a heart attack across hospital sites. In the second half of this introduction, we show how key theoretical work on boundaries and boundary objects can start to help us make sense of this phenomenon. We emphasise the dual quality of boundaries as both barrier and connector, and identify boundary workers or subjects as being instrumental in the creation of boundary objects.

### STEMI / NSTEMI patients and practices

There are two types of heart attack: ST‐segment elevation myocardial infarction (STEMI) and non‐ST‐segment elevation myocardial infarction (NSTEMI, also known in updated guidelines as non‐ST elevation acute coronary syndrome or NSTE‐ACS). STEMI has historically received more attention in cardiological research and healthcare policy and occurs when there is a complete blockage of an artery or arteries providing blood to the heart. There are relatively straightforward criteria for a STEMI diagnosis and a well‐defined management pathway to rapidly open up blocked arteries (angioplasty). There have been a range of national initiatives to increase the speed at which this patient group attend catheter laboratories where angioplasty can be carried out (Laskey *et al*. 2010) and this has resulted in decreased mortality rates (Wilson *et al*. 2013). In contrast, NSTEMI, where there is a partial blockage of an artery or arteries providing blood to the heart, is more common and has a worse long term prognosis (Darling *et al*. 2013, McManus *et al*. 2011). NSTEMI generally has a less dramatic presentation than STEMI, with no consistent electrocardiogram (ECG) changes and is more difficult to diagnose; patients tend to be older with more comorbidities (Zaman *et al*. 2014). Troponin (proteins from cardiac muscle damage) levels in the blood drive a NSTEMI diagnosis. However, because a troponin rise may also be caused by other conditions, this test still requires specialist interpretation alongside other assessments for a diagnosis to be confirmed.

Looking at heart attack mortality overall (STEMI and NSTEMI), there is significant variation in patient outcomes between different hospitals, even after adjusting for case mix (Kristoffersen *et al*. 2015, Krumholz *et al*. 2009, Seghieri *et al*. 2012). Higher survival rates have been linked to hospital‐level factors such as the volume of patients (Rasmussen *et al*. 2005, West *et al*. 2010), teaching hospital status (Patel 2007), having access to specialist cardiological care (Birkhead *et al*. 2006), higher staffing levels, and the presence of experienced inter‐professional teams (Rasmussen *et al*. 2005). Qualitative studies suggest that key drivers of quality in heart attack care include extensive interdisciplinary collaboration (Curry *et al*. 2011), especially between front‐line emergency doctors and follow‐on cardiac services (Mehta *et al*. 2006), and the presence of specialist cardiac nurses (Curry *et al*. 2011, Dunckley *et al*. 2007, Smallwood *et al*. 2009, Tierney *et al*. 2013). Inter‐professional and inter‐departmental relationships may therefore be a fruitful area for further exploration to better understand how effective heart attack care is achieved. Having established the complexity of NSTEMI diagnosis and briefly made the argument that inter‐professional and inter‐departmental relationships may be crucial to overall patient care, we now introduce boundary theory, as well as debates about its application to organisations, including hospitals.

### Boundary working and boundary objects

Boundaries are a powerful construct for understanding organisational activity because they capture the fundamental social process of relationality (Lamont and Molnar 2002, attributed to Somers 1994 and Emirbayer 1997). Relationality is central to interdisciplinary work as well as being integral to the work of categorising patients; both are needed in NSTEMI care. Hospitals are gendered and hierarchical organisations (Keshet *et al*. 2013, Mackintosh and Sandal 2010, Powell and Davies 2012), which constrains and shapes contextual boundaries within which caring work is conducted. Hsiao *et al*. (2012: 463) define boundaries as ‘a demarcation, or a sphere of activities, that marks the limits of an area, which may include knowledge, tasks, as well as hierarchical, physical, geographical, social, cognitive, relational, cultural, temporal/spatial, divisional, occupational, and disciplinary boundaries’. Boundaries can either function as a juncture or strategy for connecting two or more entities together, or as a barrier to protect autonomy, prestige and the control of resources (Chreim *et al*. 2013, Lamont and Molnar 2002).

Gieryn (1983) and Abbott's (1988) work elaborates on the concept of boundaries as barriers and markers of difference between and within professions. For example, Gieryn shows how scientists historically demarcated the field of science: from religious thinking (science characterised as empirical, objective); from engineering (science characterised as theoretical); and totally distanced science from the study of phrenology. Gieryn calls this ‘boundary work’, in which scientists promote, expand and protect their resources and autonomy. Abbott similarly described how professions seek to maintain, defend, expand and modify their existing jurisdiction. He also noted the wider system within which inter and intra‐professional competition occurs as well as greater focus on the actual tasks, work activities and services professions seek to control.

The recurrent drawing and redrawing of boundaries as professionals question each other's credibility and arenas of work occurs frequently within the healthcare context (Burri 2008, Carmel 2006, Haland 2012, Lamont and Molnar 2002, Nancarrow and Borthwick 2005). These studies typically conceptualise boundaries as conditions for separation, exclusion and as markers of difference. Haland (2012) shows that, as part of their boundary work, doctors resisted using new electronic records. The doctors associated electronic records with the taint of secretarial work and distinct from the diagnosing and treating of patients; the key to their professional identity. Nurses, on the other hand, identified electronic records as an opportunity to expand their sphere of competence and influence into a domain previously defined as the doctors’ responsibility. In contrast, Carmel showed that while occupational boundaries between doctors and nurse were maintained elsewhere in the hospital, within intensive care units it was different. In intensive care units the greater loyalty to this close ‘community of practice’ (Lave and Wenger 1991) dissolved occupational boundaries within the unit, while strengthening those same boundaries relative to the rest of the hospital (Carmel 2006).

In opposition to Gieryn's (1983) and Abbott's (1988) examination of boundaries as barriers and markers of difference, Star and colleagues argue that boundaries are useful for communication, exchange, bridging and inclusion (Lamont and Molnar 2002). ‘Boundary objects’ (Star and Griesemer 1989) articulate the ways in which actors and groups with different expertise, interests and viewpoints are able to cooperate and work effectively together. They start from the observation that consensus is rarely reached and so is not a necessary requirement for the successful conduct of work. In their example of a new museum for vertebrate zoology, Star and Griesemer showed how the different interests involved (museum director, patron, animal trappers) were able to work effectively together through agreed standardised methods (preserving animal specimens intact and proper labelling). In this arrangement, boundary objects (animal exhibits) could intersect social worlds and fulfil the informational requirements of each. Star (2010:603) argues that ‘(t)he term itself refers to an understanding of “boundary” as a shared space and “object” as something people act towards and with, so that materiality is derived from action not thing‐ness’. As either abstract or concrete, Star (2010) argues that boundary objects are most usefully understood at an organisational level. Star and Griesemer (1989) build on Latour's (1988) and Callon's (1985) notion of *interessement* and argue that the flow of objects and concepts through the network of participating allies and social worlds is an important concern.

The concept of boundary objects has been widely used in understanding organisations. In the healthcare context, Allen used it to study the implementation of a mental healthcare safety pathway that allowed clinical, managerial and service user interests to become aligned, even though it simultaneously surfaced contradictions in the pathway's multiple roles (Allen 2009). A proposed communication protocol to help overcome gendered and professional hierarchies whereby nurses can articulate their concerns to doctors in emergency situations has also been identified as a potential boundary object (Mackintosh and Sandall 2010).

More recently, it has been suggested that boundary actors may work alongside boundary objects. For example, Laine and colleagues (2016), following Huzzard and colleagues (2010), suggests that boundary subjects, such as action researchers, working in different interfaces might help to create and develop new boundary objects for other stakeholders to focus their conversations around. Boundary subjects, they argue, help make the connections happen especially through facilitating better communication. Laine and colleagues (2016:305) perceive boundary objects as vehicles that restrict as well as enable communication, whereas Star and Griesemer (1989) see boundary objects as largely facilitating communication and a space in which to articulate conflicting sets of concerns. Keshet and colleagues (2013) introduce a similar idea of boundary actors who, in their case, were trying to integrate complementary and alternative medicine (CAM) within hospital care. In one of Keshet and colleague's examples, the boundary actors (doctor and nurses trained in both CAM and biomedicine) met with other staff to propose both CAM and conventional treatments for patients’ symptoms (the boundary objects). The authors note that the main boundary actor was the doctor who was seen as effective because they bridged the two worlds of CAM and conventional medicine, had legitimacy, was trusted by other doctors and had strong personal qualities. The effectiveness of nurses as boundary actors is less clear in Keshet *et al*.'s account and they seemed to play more supportive roles. In contrast, Allen (2015), proposes that nurses are the key human mediators and translators doing the necessary work in turning patients into boundary objects.

We have examined boundary objects and boundary subjects in some detail here, because the idea of multiple actors working closely together on key areas and tasks is extremely pertinent to the care of NSTEMI patients. Likewise, the potential of boundaries to act as barriers, as suggested by Gieryn (1983) and Abbott (1988), is highly relevant to the hierarchical, constraining and spatially differentiated context of the hospital environment. Using NSTEMI heart attacks as our example, within this paper we articulate the ways in which boundary work was central to the everyday organising and coordinating work of hospital staff, and that boundary object status for NSTEMI patients was associated with better care. Beginning with the initial identification and prioritisation of patients, boundary work informed ongoing negotiations over responsibility for patients. We show how categorisation and location are crucial for the creation and maintenance of boundary objects and the parallel processes of exclusion that can lead to the marginalisation of some patient groups. We detail the essential role of cardiac specialist staff as boundary actors in the recurrent making and re‐making of patients into boundary objects and the consequences for patient care when these boundary actors were either absent or focusing on specific patient groups.

## Methods

In our ethnography we studied the care of NSTEMI patients within NHS hospitals in England and Wales. Ethnographic observations and in‐depth interviews were conducted (JH) during a two‐week period of intensive fieldwork at each site, observing work within and across emergency departments, medical assessment units (MAU), coronary care units (CCU), catheter laboratories, cardiac wards and general medical wards. The approach to fieldwork within each hospital included: (i) observation of the direct care of a sub‐sample of patients with NSTEMI from their admission through to discharge; (ii) shadowing the work of key staff identified as having a role in caring for patients with NSTEMI; (iii) in‐depth interviews with clinicians and managers; (iv) in‐depth interviews with patients and /or family members during their admission and 30‐days after their discharge; and (v) examination of patients’ medical records to clarify and provide additional clinical details (see Table 1 for recruitment and type of data collected).

**Table 1:** Recruitment and type of data collected

Type of data collection	Hospitals										Total number of participants
	1	2	3	4	5	6	7	8	9	10	
Patients observed	13	1	8	7	8	7	6	6	7	5	68
Interviews with patients (a sub‐sample of those patients observed)	7	1	6	7	8	7	5	4	4	4	53
Staff observed	36	14	15	14	21	20	26	13	20	20	199
Interviews with staff (some staff were interviewed but not observed)	13	5	12	13	17	12	12	16	21	21	142
Total hours on site observing	75	23	81	84	88	80	88	80	73	60	732

Using the Acute Coronary Syndrome (ACS) registry for England and Wales (Myocardial Ischaemia National Audit Project, Herrett *et al*. 2010), we identified a sample of 30 hospitals who were ranked either in the top or bottom third for survival rates in a 30‐day period after hospital admission. From this, eight hospitals were purposively selected: four from the top tertile, and four from the bottom, ensuring that they also represented a range of coronary interventional facilities, teaching status, volume of cardiac admissions and geography. Including two pilot hospitals (retrospectively identified as being in the middle third for 30‐day survival) there were ten participating hospitals in total (see Table 2 on characteristics of participating hospitals). The participating hospitals were aware that sampling was based on varying 30‐day cardiovascular mortality, but not where they lay in that range. In addition, the research team was blinded to hospital performance status until completion of preliminary data analysis.

**Table 2:** Characteristics of participating hospitals

Characteristic	Hospital 1 (pilot)	Hospital 2 (pilot)	Hospital 3	Hospital 4	Hospital 5	Hospital 6	Hospital 7	Hospital 8	Hospital 9	Hospital 10
Teaching status	Teaching (tertiary)	Non‐ teaching	Teaching (tertiary)	Non‐teaching	Teaching (tertiary)	Non‐teaching	Teaching (tertiary)	Non‐teaching	Teaching (tertiary)	Non‐teaching
Volume of cardiac admissions 2008 Low/Medium/High*	Medium	Medium	High	Low	High	Low	Low	High	High	Low
Type of MI patients	STEMI & NSTEMI	NSTEMI only	STEMI & NSTEMI	NSTEMI only	STEMI & NSTEMI	NSTEMI only	STEMI & NSTEMI	NSTEMI only	STEMI & NSTEMI	NSTEMI only
Catheter laboratories on site (number)	4	0	2	2	3	1	3	1	6	2**
Type of cardiology link person (total number)	Specialist cardiac nurse (1)	Specialist cardiac nurse (1)	Specialist cardiac nurse(1) + cardiologist of the week (1)	Specialist (thrombolysis) nurses (2)	Cardiac matron (1) + senior sisters (2) + junior sisters (4) + on call senior house officer (1)	Specialist (chest pain) nurses (5)	Specialist cardiac nurses (4) (not as key link for emergency department but for interhosptial transfers)	Specialist cardiac nurses (5)	Unclear /No specialist nurses (only used registrars and senior house officers)	Unclear /No specialist nurses
Number of link people on duty at any one time	1	1	2	2	4	2	2	2	0	0

Note:

*Low = 1‐249, medium = 250‐499, high= 499+, **Privately owned catheter laboratories to which we were denied research access.

Data were audio‐recorded where possible or recorded in detailed handwritten field notes. Audio‐recorded data were transcribed and field notes were written up. Patients [P] and staff [S] are numbered consecutively [P1, P2; S1, S2 etc]. Data collected by interview are indicated by [I]; data collected by observation are indicated by [OB]; audio recorded [AR]; and field note recording only is [FN]. The study received NHS Research Ethics Service approval (10/H0107/75).

All data‐sets were analysed thematically and a framework was used to organise the data and to identify key themes (Miles and Huberman^1994^) and concepts (Glaser and Strauss 1967). We explicitly used multiple perspectives (patient, clinical, managerial) to inform the development of our analysis to identify local organisational and broader structural conditions (Corbin and Strauss 1990), which might influence the care of patients classified as NSTEMI. Emerging concepts were developed initially by JH and HC and in collaboration with team members (KF, GF, ME, CD and RJ). Findings and themes were reviewed by the multi‐disciplinary research team including clinicians, cardiologists, cardiac nurses and patient representatives. We referred to the English National Institute for Health and Care Excellence (NICE) guidelines (2010, 2013) to benchmark the quality of NSTEMI care.

## Findings

We found that boundaries were central to NSTEMI patient care. Beginning with the initial categorisation and prioritisation of patients, boundary work extended into negotiations over responsibility for patients as they were transferred and admitted to different wards, and entered different domains for diagnostic tests and treatment, such as within catheter laboratories. Importantly, in order to navigate boundaries successfully, a patient needed to become a stable boundary object. Once stable as a boundary object, patients were more easily recognised by staff and by the wider hospital system, and all the necessary coordinated activities and routines of different professions and specialists across different sites were more likely to occur. Classification played a significant part in becoming a successful boundary object and the diagnostic and categorisation process was an important first step to a patient being ‘worked on’. Difficulties and uncertainties in classification, were key reasons why many NSTEMI patients faltered as boundary objects, with a significant impact on their access to care. NSTEMI classification and categorisation is also moral work and some patients were denied boundary object status, which in turn excluded them from mainstream treatment. Value placed on youth, urgency and clarity tended to disadvantage older and more complex patients, which in turn impacted on NSTEMI patients in general (an older patient population with more comorbidities, who are more difficult to diagnose). The creation of NSTEMI boundary objects draws attention to what or who gets left behind, the parallel processes of marginalisation and the importance of location. Nurses and specialist staff could play a key role in doing boundary work and helping to enact NSTEMI patients as boundary objects. Within some sites, boundary work on behalf of patients included breaching silos, harmonising spatial, disciplinary and hierarchical boundaries to guide NSTEMI patients through the hospital system. Without boundary actors helping to champion NSTEMI patients and enact them as boundary objects, the hierarchical and spatially fragmented hospital environment remained constraining and dominant, distorting patient care and access to care and outcomes.

### **Categorising patients: who crosses the boundaries into acute cardiac care?**

An important initial task for emergency staff and general medical doctors was to triage patients arriving in the emergency department. Patients presenting with chest pain needed to be identified as likely cardiac patients and NSTEMI patients needed to be identified as being possible NSTEMI patients. Patients already admitted to the hospital, but not necessarily to the cardiac wards, could also experience new onset chest pain and processes needed to be in place for staff to recognise and interpret these symptoms.

### Complexity of NSTEMI diagnosis

Because there is no simple non‐invasive test that can determine whether a partial blockage in an artery has occurred, medical teams must take into account and assess a range of factors to make an NSTEMI diagnosis. We observed that this included asking the patient to describe their chest pain, taking an ECG, and carrying out repeat troponin tests. One cardiologist described how the complexity of a NSTEMI diagnosis continually challenged their specialist expertise and could not be neatly worked out using simple diagnostic tools such as flowchart diagrams:

I think it's really hard to diagnose NSTEMI … you'll get people who will come in who have got troponin at 0.06 [consistent with no damage to the heart], you take them to the lab and they've got terrible three vessel disease and a blocked vessel. You have someone who's got a troponin level of two, you take them to the lab and they've got no occlusions [blockage in arteries]. There's a huge emphasis in my view, in taking a good history and putting it in clinical context. Pathways and flowchart often skip that because it's very hard to describe … [Pathways say] please use for suspected cardiac pain. It doesn't tell you how to suspect cardiac pain … is it chest tightness? How long does it last all these kinds of things, they're rather spurious. You have to take the history and get the whole combination (S1, cardiologist, Hospital 6, I:AR).

While a key characteristic of many NSTEMI trajectories was persistent diagnostic uncertainty, as well as decisions about which patients might be NSTEMI patients, judgements needed to be made about severity and stability, who to prioritise for more intense monitoring, which follow‐on ward might be appropriate and under which specialty. For a significant proportion of cases, medical teams had to negotiate the added complexity of assessment of whether urgent treatment needed to be given. According to NICE guidelines, NSTEMI patients assessed at medium to high risk of further poor oxygen supply to the heart muscle or of complications should be offered a coronary angiogram within 96 hours (NICE 2010), although there is still uncertainty about the benefit of an early invasive strategy even in high‐risk patients (Jobs *et al*. 2017).

Because diagnosis and categorisation for NSTEMI patients was a complex and often iterative process, cardiac specialists needed to help non‐specialist clinicians and be available to teams in the emergency department. However, we found that poor working relationships between cardiac and emergency staff often hampered collaboration. For example, a senior nurse within one emergency department expressed her frustration with the lack of access to cardiology consultants. She described how the cardiologists stayed within their domain (their offices) rather than cross the physical boundary and attend the emergency department (the shop floor) and suggested that this was the result of doctors safeguarding their workload and also a lack of leadership from senior cardiologists:

We don't feel as though anyone fights our corner for us, we are all a bit battle weary … [cardiac consultants] do the bare minimum … [and] the clinical lead we feel has no clinical credibility … It is a huge leadership issue … of course you've got office stuff to do and there will be HR stuff to deal with, but they have to have some clinical credibility and be able to nip in and out and advise … everyone works very much in silos … by nature in A&E we're jack‐of‐all‐trades, masters‐of‐none and there isn't that sort of advice line really and then we get the blame for referring everything or because we haven't got the confidence to necessarily discharge them … years ago you could ring a consultant up … ‘I've got this patient, can you just cast your eye over them, I just want a bit of advice’ and they'd say ‘yes I'll pop down’. That has completely gone … we get fed up having to go and find them in their offices, they are meant to be on the shop floor (S2, senior nurse, Hospital 10, I:AR).

Due to the potential complexity of diagnosis, NSTEMI patients were immediately at a disadvantage. They often required non‐specialist staff to be on the lookout to identify them, to suspect NSTEMI, to pay attention, wait, seek and secure advice. For many patients, only with this sort of active monitoring and scrutiny, could the NSTEMI diagnosis start to evolve and be made. Specialist cardiac staff were usually based elsewhere and so good interdepartmental links were essential and had to be established and maintained in order to support the diagnostic process. As well as spatial boundaries (separate emergency and cardiac departments), hierarchical relationships between nurses and doctors obstructed effective interdepartmental working. Not necessarily having the specialist or diagnostic training required, emergency staff and nurses needed to overcome hospital hierarchies to obtain the advice and permission they need. Needing to ‘fight’, the absence and inaccessibility of cardiologists and the perceived priority given to office‐based work is, to some extent, suggestive of Gieryn's (1983) and Abbott's (1988) work on the erection of barriers to protect autonomy, prestige and the control of resources. The contexts in which NSTEMI patients might be identified and begin to emerge as boundary objects, worthy of greater attention, was riven with professional, hierarchical and spatial obstacles.

### Applying diagnostic sub‐types or broad classification to potential NSTEMI patients

Partly because diagnosis was not straightforward for NSTEMI patients, there was a wide variation in diagnostic practices across the sites and a diversity of staff involved in those processes (see also Table 2 for more information on the type of link staff in each hospital). Some of our hospital sites applied additional and restrictive diagnostic categories that delayed the timely identification of NSTEMI patients. In others, a lower diagnostic threshold resulted in large numbers of patients, many with a low risk of NSTEMI being referred to specialist cardiac services. For example, cardiac nurses within Hospital 8 were required to apply an additional diagnostic category that meant they had to further classify each patient within a sub‐type of NSTEMI (type 1 and type 2). Medical teams typically disputed this additional categorisation, because it was a harder diagnosis to achieve definitively and thus this requirement typically delayed diagnosis and the transfer of patients to a specialist cardiology team:

These type 2s are a nightmare … is it a type 1or is it a type 2? Not always that clear cut, … we're just … holding off until we've got that final diagnosis … one of our consultants might think it's a type 1 MI, then somebody else might think it's a type 2. We do get disagreements … for us as nurses, what that sometimes means is that we hold off and hold off and hold off until the patient's under the consultant (S3, senior nurse, Hospital 8, I:AR).

While the cardiologists in Hospital 8 praised the cardiac nurses’ work in helping to identify NSTEMI patients, they resisted becoming involved in this type of work themselves, preferring to save their expertise for the ‘genuine’ and ‘proper’ cardiac patients. However, they did not seem to appreciate how important taking a more active role in helping to identify and categorise patients might be:

The perfect situation would be … the full cardiology team on call, enough staff to see patients at the front of the door … [But] if you spend your time chasing up all the referrals that takes you away from the genuine acute proper cardiology cases (S4, cardiologist, Hospital 8, I:AR).

In contrast, large numbers of patients received the broad classification of ‘possible heart attack patients’ within the emergency department of Hospital 9. This resulted in large numbers of admissions to acute cardiac wards and this in turn, caused an overload in the cardiac services. Staff felt that this situation had been caused by the lack of explicit agreement between emergency department staff, MAU staff and cardiologists on the diagnostic threshold for transferring a patient or when cardiac specialists should be called on to assess patients. One cardiologist described this:

Assessment of NSTEMIs is at the moment a little bit crumpled … an initial triage, yeah and that might help a little bit more … we can be a bit more prescriptive in terms of who ought to get them but at the end of the day, A&E [accident and emergency] are not going to be in a position to necessarily help, they just send it [patients] up and so it's up to us to try and get our processes in order… the sheer number, the economy of scale has been lost within numbers and the pressure (S5, Hospital 9, I:AR)

Working relationships form around boundary objects where a shared syntax or language is established and different communities of practice can represent this knowledge, communicating concerns and questions (Fox 2011). While boundary object theorists generally agree that full consensus is not required for different parties to work together around a boundary object (Star and Griesemer 1989) there does need to be a minimal recognition and understanding of the identity of a boundary object. For many NSTEMI patients, their diagnosis emerged slowly, often needing greater interpretive work (Allen 2015) and typically only considered to be definitively revealed once an invasive diagnostic angiogram had been carried out in the catheter laboratory. It was therefore not always obvious to staff which patients they needed to focus their attention on. This also meant that staff could not easily start the medical monitoring and treatment specific to NSTEMI care. Being required to assign additional underlying causation (type 1, type 2), not applying strict enough criteria for admission, as well as poorly maintained relationships between cardiac specialists and emergency staff, all created obstacles to starting the NSTEMI diagnostic process. However, additional obstacles also delayed staff being able to work effectively together on and around the NSTEMI patients as boundary objects.

Diagnosis is a key organising feature of hospital work and without a clear patient identity it was hard for staff to manage and co‐ordinate their care. Being a patient population without a clear diagnosis for a substantial period of their admission, meant that NSTEMI patients were at a significant disadvantage. In these examples, nursing knowledge, jurisdiction and lack of autonomy meant that nurses were reliant on the involvement of senior doctors in diagnostic decisions. This reliance disrupted nurses’ ability to carry out key follow‐on diagnostic related tasks and the enactment of these patients as boundary objects. Early and stable classification was an important and initial entry point towards boundary object status. As well as classificatory hurdles hindering initial entry towards boundary object status, strongly defended professional boundaries and protection of tasks and resources remained a significant feature of the constraining clinical context.

### *Judgements about who to prioritise for angiography*

In all hospital sites we observed that the diagnosis and treatment of STEMI patients were consistently prioritised over NSTEMI patients. This meant that NSTEMI patients had restricted access to treatment and interventions (within the catheter laboratories) and specialist wards such as the CCU where their symptoms could be more intensely monitored:

[The STEMI service] on occasions works to the disadvantage of NSTEMIs in terms of cath lab slots available, so the NSTEMIs wait time has to suffer … [and] because of the high number of STEMIS, primaries, that tend to require the CCU observation afterwards, it restricts where the NSTEMIs can be initially positioned (S5, cardiologist, Hospital 9, I:AR).

STEMI patients are thus revealed as having clear boundary object status. As boundary objects, STEMI patients are both objects and shared spaces (Star 2010) that a variety of different classes of hospital staff, across locations and specialisms, and with different perspectives, must act towards to achieve care. In contrast to NSTEMI patients, STEMI diagnosis was generally straightforward. Allen (2015) argues that categorisation and patient identities are useful for everyday organising and routines. With the ability to receive a quick and uncomplicated diagnosis, STEMI patients were ‘barn door patients’ which Allen (2015:89) describes as occurring when patterns of signs and symptoms point to well‐known clinical conditions and trajectory pathways that enable staff to organise around. Being able to be rapidly categorised and identified, meant that the management and treatment of STEMI patients could follow straightforward, uncontested protocols. Across all sites it was well recognised that STEMI patients needed swift action and immediate transfer to a catheter laboratory for percutaneous coronary intervention (also known as angioplasty). Value is placed on conditions that are amenable to protocols (Berg 1997) and conditions requiring urgent action (Jones and Hall 1994, Moskop and Iserson 2004). National initiatives focusing on improving time to angioplasty for STEMI patients reinforces this prioritisation (Laskey *et al*. 2010). STEMIs’ boundary object status has therefore arisen over time from durable cooperation among different communities of practice and retains a common identity, whilst still being adaptable to local needs (Bowker and Star 1999). Cooperation around boundary objects is facilitated through agreed standardised methods, which, in Star and Griesemer's (1989) case of a new museum, were intact animal specimens and proper labelling. Being amenable to simple and uncomplicated classification and subject to straightforward protocols of action, were the method of standardisation which helped the creation of STEMI patients as stable boundary objects.

There were clear rewards and positive consequences from having a strong boundary object status. In this case it meant STEMI patients were consistently prioritised for treatment in the catheter laboratory and were more likely to be admitted within intensively monitored wards such as the CCU. With only a certain number of slots in the catheter laboratories for diagnostic work and treatment, STEMI patients were NSTEMI patients’ constant competitors. STEMI prioritisation reveals the relational aspects of both boundary object creation, and its impact on the marginalisation and sidelining of other groups.

Although conceptualising patients as objects is ethically uncomfortable, others have suggested patients may usefully be seen in this way (Featherstone *et al*. 2006, Lindberg and Czarniawska 2006, Williams *et al*. 2008) and this interpretation is consistent with our data. Apart from patients being asked for an initial description of any chest pain (and very occasionally discharging themselves from hospital) there was little evidence of STEMI or NSTEMI patients exercising agency, which is arguably similar to the experiences of many patients undergoing acute hospital care (Laird *et al*. 2015).

### NSTEMI patients excluded from the treatment pathway

Although NSTEMI diagnosis often took time to emerge, some patients with a definite NSTEMI diagnosis did not receive any specialist cardiac treatment and in some hospitals, whole categories of these patients were excluded from the NSTEMI pathway. For example, two hospitals had explicit policies allocating NSTEMI patients aged over 84 years to a different treatment pathway. In Hospital 5, patients aged 85 and over were routinely excluded from cardiology services and placed on medicine of the elderly wards, which meant that they did not have access to a review or treatment by the cardiology team unless they were judged by the cardiac specialists as particularly ‘bright’ or already known to their service (OB:FN). For example, one 87 year old patient arrived in the emergency department with chest pain and shortness of breath:

She was given an NSTEMI diagnosis and transferred to a medicine for the elderly ward. She continued to have chest pain but was not reviewed by a cardiologist because the cardiology team did not see patients admitted to that ward. After waiting seven days for a cardiac ultrasound to investigate for heart failure, she asked to be discharged. She returned home and died five days later (P1, Hospital 5, OB:FN and medical notes).

Staff indicated that not seeing a cardiologist and being placed in the medicine for the elderly ward was not an uncommon experience in this hospital for people over 85:

Spoke to senior cardiac nurse, who said age and co‐morbidities are factors ‘it's very hard, but it's how it is’. I ask about the patient I have recruited and why she has not seen a cardiologist. The nurse replies: ‘whoever was on at weekend decided they didn't want to see her. At 87, that might happen. You have to think of their whole situation and sometimes it's best to keep them with medicine for the elderly and treat medically and not to intervene aggressively’ (Hospital 5, OB:FN).

Categorisation, Bowker and Star (1999: 5) argue, is also moral work and ‘each category valorises some point of view and silences another’. While diagnosis was often a crucial first step towards being clearly recognised and becoming a potential boundary object, it was not the only driver. As well as diagnosis, patient identities were affected by a patient's age or, rather, a perception of their age. These data highlight more clearly both the consequences of becoming or not becoming a boundary object and the moral work involved in those categorisation decisions. Star and Grieseme (1989)r also stress that the choice of clientele and personnel is important in the flow of (boundary) objects and concepts through a network of participating allies and social worlds. In some hospital sites, decisions were made that meant older patients did not receive the same type of attention and treatment as younger patients and so were excluded from the possibility of becoming boundary objects. In these hospitals shared meanings, values and beliefs had come to agree that patients over 85 years old were less appropriate or ‘deserving’ of (Grimley Evans 1997, Jeffery 1979, Nugus *et al*. 2010) certain forms of care. This is arguably another example of boundary object marginalisation. However, questioning overly aggressive treatment in older patients might also be seen as positive (Kaufman *et al*. 2004): it is possible that different sorts of boundary objects were simultaneously being created in other departments such as medicine for the elderly departments with a different set of values.

### ***Holding*,* passing and falling between responsibilities and spaces***

**Figure 1:**
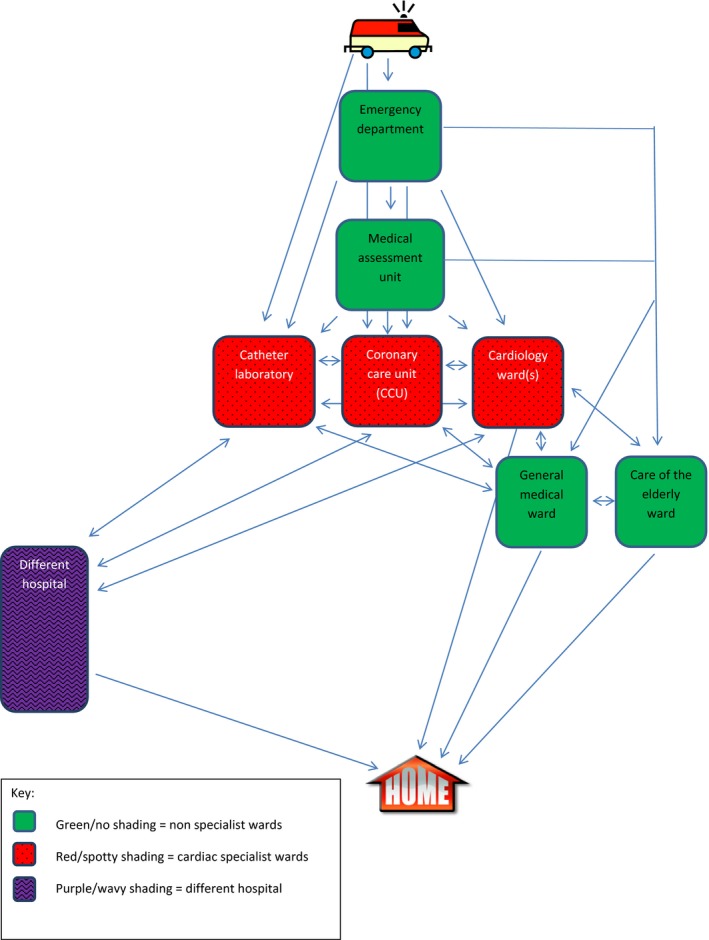
Possible pathways through hospitals for NSTEMI patients

Patients classified as NSTEMI (or possible NSTEMI) needed inpatient admission; therefore bed allocation, resources, treatment and clinical teams all required coordination and alignment, sometimes sharing this work between different hospitals with different facilities and capacity for treatment (see also Table 2). This coordination work was primarily carried out by nurses (see also Allen 2015, Deaton *et al*. 2016). It was further impacted by ongoing diagnostic uncertainty, severity and stability of the patient's condition and presence of comorbid conditions. All of these factors affected the process of patient transfer across teams as well as ward and hospital boundaries. The multiple possible pathways through the physical spaces of a hospital for NSTEMI patients are indicated by Figure 1.

### Territory and responsibility within wards

We identified that NSTEMI patients were sometimes admitted within dual‐condition wards, where the physical spaces of wards, their bays and beds, were dispersed between a number of different clinical specialties and medical teams. However, this caused uncertainty for clinicians and medical teams about their patient caseload, lines of responsibility and accountability. When clinical teams from different specialisms had responsibility for patients within the same wards, then (sometimes intense) negotiations were needed to agree the identity of a patient's clinical team. The ward teams constantly negotiated the boundaries between clinical teams and the boundaries of the patients’ location within the ward and who had, or should have, ownership of the patient. For example, in Hospital 4, the nursing staff within a new mixed cardiac and respiratory ward reported that making this dual‐condition ward system work (and keeping the patients aligned to and reviewed by an appropriate medical team) was taxing:

We've got obviously a cardiac patient in a respiratory bed so the respiratory consultant's done his ward round today but he won't see her because she's a cardiology patient … [And the cardiology team] It's not their referral … we're constantly chasing the doctors asking who does this patient belong to? And who's going to see her? (S6, nurse, Hospital 4, I:AR)

This mix of caseload within the wards had consequences for patient care and several of the patients observed in this ward experienced treatment delays and omissions, with consequences for patient care:

An 82 year old woman was waiting for a specialist review by the cardiologists attending this ward. However, after eleven days as an in‐patient in the ward she was discharged without having had the angiogram planned for her. A ward nurse confirmed that this patient had been missed by doctors on some ward rounds because there had been a disagreement over who had responsibility for the patient within this dual condition ward. The patient was interviewed three weeks after discharge and she reported that she had no contact with the rehabilitation team and was missing two of the medications she was supposed to have on discharge (P2, Hospital 4, FN:OB and medical notes).

In dual‐condition wards, the NSTEMI patient's identity as a boundary object faltered. In the absence of clear agreements, patients in the ‘wrong bed’ were overlooked on ward rounds. The nurses here were struggling to coordinate, facilitate and enact an effective work‐around for these NSTEMI patients to be recognised as boundary objects. Classification once again was the dominant organising principle within hospitals for the creation of boundary object status, however, location mattered too. In line with Gieryn's (1983) and Abbott's (1988) work on the preservation of professional boundaries, the doctors seemed unable or unwilling to see patients whose diagnostic categorisation did not align clearly with their own specialisation (cardiac or respiratory). In response, the nurses’ boundary work involved directly trying to resolve these disputes between specialties; but they had limited authority to influence and insist on doctors’ involvement (Allen 2015:76). As this and other studies have shown, hospitals are intensely hierarchical organisations where power and status derive from a combination of gender, profession and organisational roles (Keshet *et al*. 2013, Mackintosh and Sandal 2010, Powell and Davies 2012). These descriptions of hierarchical relationships hindering some forms of collaboration correspond well with Clegg's (1989) notion of the common constraining and hierarchical ‘episodic circuits of power’. Clegg argues that most power relationships within organisations are in the form of episodic circuits. Prohibitive rules are a significant feature of episodic circuits of power and the relationships are characterised by constraint, resistance and reluctant compliance.

### Negotiations between hospitals

Boundary negotiations over territory, specialism, responsibility and workload also operated on a regional scale. A significant proportion of NSTEMI patients needed to be transferred between hospitals to receive appropriate care, for example, because they needed a diagnostic angiogram or angioplasty in a catheter laboratory. Whilst we observed significant difficulties with the transfer of NSTEMI patients, this was not the case for STEMI patients who were typically transferred smoothly between hospitals. Larger tertiary hospitals seemed to have greater power to decide whether a patient could become part of their caseload or remained with the smaller district hospital and also whether they would accept patients from or transfer them back to a district hospital. Hospitals had treatment goals such as the 96 hour angiogram target and, where information was forthcoming, payments seemed related to number of patients admitted or number of procedures carried out. Controlling the flow of patients was therefore important economically and bed managers were also keen to maintain the space and flexibility for the treatment of urgent cases. Thus, patients who could be discharged quickly were the preferred population. More complex patients (who tended to be NSTEMI patients) generally required a longer stay and took bed spaces that more straightforward patients could utilise more ‘efficiently’.

There were ongoing negotiations for NSTEMI patients to access services across territories. One doctor within a district hospital had difficulty trying to secure a bed and a slot in the catheter laboratory at the local tertiary hospital for a NSTEMI patient with additional complexities:

The cardiologist comes from the catheter labs early in the morning. He is a bit irritated because the tertiary centre has said they cannot take his patient. But there is a patient here that needs an angiogram and haemodialysis. There is no haemodialysis team in this hospital, only at the tertiary centre. The cardiologists says he will have to try harder to persuade the tertiary to take him. (Hospital 6, FN:OBS)

In another example, a senior nurse in a tertiary hospital explained their planned transfer of a patient back to a district hospital and suggested that responsibility for the patient remained with this smaller district hospital. The nurse describes a number of techniques that she could employ to coerce this hospital to accept the transfer, including enroling the cardiology team to lead the negotiation, and suggesting that the patient would be transferred regardless:

We are very reliant on [district hospitals] giving us the bed. Now what usually happens is I'll get notified that they are not taking a patient back from coronary care, and I will tell coronary care to contact them again … ask them where they would like us to send that patient? Which is a different question. It's we are sending the patient … do you want us to send the patient to a ward or do you want us to send him or her to A&E? … but the decision is theirs because it is their patient (S7, senior nurse, Hospital 9, I:AR).

A clear community of practice (Lave and Wenger 1991) and agreement to work together and transfer STEMI patients smoothly between hospitals was facilitated by their intact and stable boundary object status. However, for NSTEMI patients, greater efforts and negotiations on their behalf were required for them to become accepted for treatment and to control the timing of their return. Transfer and coordination work was mainly carried out by nursing staff (Allen 2015), and nurses seemed to feel the burden of patient responsibility most keenly. Categorisation and location continued to matter and to influence the enactment of NSTEMI patients as boundary objects. The work of enacting NSTEMI patients as boundary objects was undertaken by both nurses and doctors and, despite the need for interdependence between facilities (Nugus *et al*. 2010), larger hospitals could take greater advantage of spatial boundaries in the effort to protect and control their resources. Even when a clear NSTEMI diagnosis had been achieved, because NSTEMI patients were more likely to have additional comorbidities, they rarely aligned neatly within straightforward treatment protocols (Berg 1997). In these ways NSTEMI boundary object status remained more vulnerable to interruption than for STEMI patients.

### Marginal locations

Nursing and cardiology teams’ responsibility for NSTEMI patients typically fell away at the ‘edges’ of hospital care, especially during the discharge process. From observations, this group of patients seemed more likely to be transferred to a discharge lounge, which did not have permanent medical teams attached to them. For example, two hospitals used discharge lounges to help relieve the pressure on the cardiac wards; patients were placed here prior to discharge while waiting for medications and paperwork. In Hospital 6, the relatively new discharge lounge had 32 mixed chairs and beds and, despite some staff claiming that NSTEMI patients were never placed in there, one patient was observed in the discharge lounge. A nurse discussed her concerns about placing cardiac and other vulnerable patients in a setting meant for medically fit patients and, on occasion, she describes resisting pressures to place patients in unsafe ‘marginal’ places:

We shouldn't really have cardiac patients because we don't have the doctors or the facilities … in theory people should be coming to us medically discharged but that's not true … when we first had this [it] was great because you just thought at least you've got somewhere you can put patients on stretchers … in fact on one occasion … I am down to the site manager and just said ‘I am sorry but I am going to have to tell you that I am not opening a discharge lounge, we have inpatients and we don't have the staff to do it’ … it is not safe yes they are medically fit to go home, but a lot of them might become confused, have mobility problems, still need taking to the bathroom. They still need the care (S8, nurse, Hospital 6, I:AR).

Spatially related boundary work was constantly required to negotiate admission within wards, appropriate bed allocation for patients, and transfers between wards and hospitals. This was particularly the case when spaces had been given a relatively new designation such as dual condition wards and discharge lounges, created by institutions in response to pressure on resources and increasing patient numbers. In these marginal locations, clinical responsibility for patients was typically unclear or still in the making and there was a continual negotiation between ward staff, professions and specialisms about the role and remit of these wards and where responsibility and accountability for patients rested. This uncertainty potentially risked the quality and intensity of monitoring and review available for NSTEMI patients placed within them. Location sometimes undermined an NSTEMI patient's potential for boundary object status and NSTEMI patients (rather than STEMI patients) were more vulnerable to being placed in marginal locations.

### **Close collaborations and ambiguities between specialisms**

### Patient fragmentation

Cardiology teams recognised the challenges of the physical/spatial boundaries of caring for their patient caseload across wards and between other specialties. Some hospital systems responded to these spatial boundaries by trying to stretch the expertise of a small number of specialist cardiac nurses more widely across the hospital. One way to do this was through remote telemetry. Remote telemetry separated care for NSTEMI patients between two groups of nurses, with ward‐based staff responsible for the care of the patient at the bedside and cardiac specialist nurses elsewhere responsible for the patient's heart monitoring. In Hospitals 4 and 10, NSTEMI patients admitted to non‐specialist wards had their cardiac activity monitored using remote telemetry, with the ECG monitor screen located at a distance from the patient. This system of remote monitoring was a response to the increasing caseload and the training gap of nurses in other wards who were not able to interpret the ECG monitoring of patients in their wards:

We installed telemetry throughout … and because of the busyness of the hospital and educating such a wide range of people to just, basically to understand a few simple rhythms I thought let's keep it focused in the coronary care unit where we've got dedicated staff who are trained, it doesn't mean to say I don't want to train staff on the ward … but let's focus it where the experts are (S9, senior nurse, Hospital 4, I:AR).

While remote telemetry was thought by some to be in the best interests of patients, the specialist nurses who were responsible for monitoring these patients, were typically based within the CCU, which meant their responsibility for caring for high dependency patients who required continuous monitoring and treatment, was further stretched by monitoring and responding to the remote telemetry alarms. These systems did not take into account the challenge of communication across sites and competing priorities within wards:

If you cannot spare anybody to go running off without endangering the [CCU] unit, then you have to keep going through the channels of phone calls … and looking at a monitor of a patient in real time that you can see is getting progressively worse, in front of your eyes … you know there's nothing you can do … That patient would be dying … you start phoning the wards. If you can get through straight away, brilliant. But there have been a couple of occasions where you are phoning against an engaged signal on all the numbers you know (S10, senior nurse, Hospital 4, I:AR).

CCU specialist nurses were limited to calling the ward staff if they wanted to report the changes in a patient, and seeking advice and direct communication with doctors was actively discouraged. The senior nurse who introduced the remote telemetry in Hospital 4 conceded that the system was highly dependent on good communication. Although the telemetry system continued to monitor the patient, the patient may be transferred to other sites of care within the hospital. Safety and efficacy was thus sometimes undermined by the difficulty of locating the patient and the flawed lines of communication between wards:

There's some issues where they've transferred a patient from one ward to another and the wards haven't contacted us and told us, so then if there was an arrhythmia we would obviously go to that ward and we wouldn't know where the patient was … we had a couple of incidents about this last week (S9, senior nurse, Hospital 4, I:AR).

Splitting responsibility for patients between staff in different locations across the institution, through the use of telemetry, undermined the potential for NSTEMI patients to be enacted as boundary objects. Although a seemingly minor point about preserving animal specimens intact as part of the standardised methods that are key to working together, Star and Griesemer (1989) may have been making a more fundamental point about the importance of internal integrity and wholeness in the creation and maintenance of boundary objects. The split responsibility for patients’ bodies through remote telemetry demanded a high level of ongoing communication between teams in different locations. Rather than helping to bind different wards and different professional roles into working together on patient care, the use of remote telemetry seemed to reinforce ward loyalties and barriers between groups and professions based on knowledge and roles. CCU and ward nurses struggled to be fully focused on the NSTEMI patients being monitored using remote telemetry, with CCU staff's primary responsibility for their CCU patients (and who were more likely to be STEMI patients) and ward staff who said they did not feel the same sense of responsibility for the patients monitored via telemetry. Limiting telemetry expertise to a few specialists was an explicit strategy reported by the senior nurse in Hospital 4 and consistent with the ongoing theme emerging from our data about boundaries becoming barriers to protect and control resources both between and within professions (Abbott 1988, Gieryn 1983). That the nurses in this example were prevented from direct communication with doctors reinforces a second key theme about the constraining and traditional power structures within which hospital staff typically operate.

### *Collaboration*,* control and dependency*

Another area of intense boundary negotiations played out within a specific hospital territory was when cardiac specialists depended on the activity of staff from other disciplines for NSTEMI patient diagnosis, and to provide treatment and interventions. However, to achieve this, different expertise and different ways of working must be aligned within the same space with a shared recognition of urgency and importance. The majority of the cardiology teams described the difficulties in working with radiographers or radiologists who provided essential support in the catheter laboratories where angiograms and angioplasty are carried out, but who also had their own (non‐cardiac) caseloads. For example, one cardiologist elaborated on his frustrations with radiology staff; he believed their overall control of the catheter laboratory critically affected his team's ability to carry out procedures that he considered essential for NSTEMI patients:

The physical situation of where the lab is counts as the radiology department … the radiology gets to call the shots on what's happening on the ground, um, which is potentially difficult for cardiology because there are many processes down there that could be better … but we don't have any direct way to influence it (S1, cardiologist, Hospital 6,I:AR).

A senior nurse in Hospital 6 echoed this and described her experience of other specialisms making decisions about which patients to prioritise in isolation; emphasising that their ability to exercise any influence was impacted by the disciplinary location of the treatment:

I got this call from one of the radiologists to say ‘I've delayed the cardiac lab service because we've got too many acutes’ for vascular … all the time it's, cardiac takes second preference down in that lab … In the old building we had our own day case unit within cardiology and we could manipulate things because we have some control (S11, senior nurse, Hospital 6,I:AR).

An exception however to these commonly reported difficulties was in Hospital 10, where the catheter laboratory service was provided by a private company who responded flexibly to demand:

We've always found [company name], for obvious reasons, flexible because as an organisation they gain from doing more work. So if we have cases to do at lunch time or heading into the evening then in the past they've been very flexible about accommodating that … there's clearly a bonus element I think in it for the staff (S12, cardiologist, Hospital 10, I:AR).

The creation and maintenance of STEMI and NSTEMI boundary objects was largely played out within the domain and reach of cardiology. But in the catheter laboratories, boundary object patients, championed respectively by different specialist teams, came into direct competition with each other. Radiologists and cardiologists both sought to exert control over the activities carried out within the catheter laboratories and were further examples of disciplinary boundary disputes (Abbott 1988, Gieryn^1983^). The location of the laboratories seemed key to undermining cardiologists’ influence on the work done there. Allen (2015) has also noted that patients are in competition with each other for access to services, facilities, time and attention and that nurses regularly mediate between the needs of individuals against the needs of the organisation in serving the whole population (see also Davies *et al*. 2000). In the catheter laboratory it was unclear which patient populations had priority: radiologists could claim to represent the whole hospital population whilst cardiologists, by virtue of their specialty, would focus on the cardiac population and their particular needs for urgent intervention. As more stable boundary objects, STEMI patients generally took priority over NSTEMI patients. Not being able to secure a timely slot in a catheter laboratory could delay a more definitive diagnosis for NSTEMI patients, severity assessment, and treatment and delays in discharge that could further limit the availability of bed spaces for other patients. Financial rewards sometimes helped support capacity in the catheter laboratories and therefore the maintenance of boundary objects. The neat mapping of disciplinary jurisdictions onto particular spaces (Prior 1988) was contentious in the catheter laboratories. The catheter laboratory is a key moment in the NSTEMI patient trajectory where disciplinary boundaries met and negotiations for their care were influenced by territorial, hierarchical, economic and professional axes. The catheter laboratory is also a key moment for the disruption or maintenance of boundary object status for all cardiac patients.

### **Dedicated boundary negotiators**

### Cardiac specialist nurses

Specialist cardiac nurses were employed within eight of the 10 hospitals (see Table 2) and these posts signify that hospitals have recognised some of the particular challenges in caring for NSTEMI patients. However, the delineation of specialist cardiac nurse roles differed markedly between the hospitals: in some they played a central and enhanced role across the hospital such as being able to prescribe medication and reduced reliance on the cardiology team; whilst in other hospitals they had more narrowly defined tasks such as data recording and monitoring/transfer.

In Hospitals 1 & 3, specialist cardiac nurses played an enhanced role in negotiating across physical/spatial, hierarchical, professional and specialist boundaries. For example, one specialist nurse described how they identified patients, led NSTEMI patient care and helped to negotiate the patient pathway through the hospital:

I try and catch people down in A&E and [MAU] before they go to the wards … I go through the diagnosis and look to see if there's anybody with chest pain … [emergency staff] they'll catch me and say … I've got this patient on 5b I'd like you to have a look for me … mopping up any loose ends as well because often they're seen by junior doctors … I have a very good relationship with the cardiology consultants … I'm always diplomatic… I suggest things in a way that the decision is made that I see, I see as appropriate but I, I don't like to use the word manipulate, but I'd like to sort of make the doctors think it's their idea … and getting what I want for the patient … And if tests are needed then I will expedite those tests. …. It's all about being proactive. … I can get things moving (S13, nurse, Hospital 3, I:AR).

With support of cardiac specialist nurses, NSTEMI patients could emerge as successful boundary objects. This is consistent with the notion of boundary objects shifting in status in different settings or over time between those that are successful and positive or inhibitory, negative or failures (Bowker and Star 1999, Fox 2011, Thomas *et al*. 2007).While most staff were, to some extent, regularly engaged in various sorts of boundary work, cardiac specialist nurses were key boundary workers, actors (Keshet *et al*. 2013) or subjects (Laine *et al*. 2016) coupled to and dedicated to the creation and maintenance of boundary objects. Cardiac specialist nurses played a significant role identifying possible NSTEMIs, where diagnosis and categorisation has been highlighted as a key initial stumbling block in the transformation of patients to boundary object status. The specialist nurses worked specifically on raising NSTEMI and possible NSTEMI patients into view, recreating them as more stable and visible boundary objects, worthy of others’ input. They did this by physically searching out potential NSTEMI patients in the emergency and other departments, smoothing communication between different specialisms and levels of seniority, and speeding up action when necessary. They were highly trained and could carry out ‘extra’ tasks such as prescribing, which has traditionally been considered the territory of doctors (Avery and James 2007). The nursing roles often included examples of local tailoring which Star (2010) argues is back stage work, important in the development of boundary objects but which may be less visible to the participants involved. Star and Griesemer (1989) also draw attention to the flow of objects and concepts through the network of participating allies and social worlds and stress that the process of management across those worlds includes crafting, diplomacy, the choice of clientele and personnel. Crafting and diplomacy are clearly present in this account of activities and the personnel to do this work; the cardiac specialist nurses.

In Keshet and colleagues’ (2013) definition, boundary actors denoted the individuals who mediated between incommensurable paradigms in the context of power inequalities. From the example given in this study, and the emphasis on such skills as diplomacy, cardiac specialist nurses clearly operated in the context of power inequalities and their role is not dissimilar to that of ‘facilitatory circuits’ of power. Clegg (1989) argues that out of the more usual constraining and hierarchical episodic circuits of power, facilitatory circuits may sometimes emerge. Facilitatory circuits are where the normal rules of organisational practice may be altered and authority is delegated to groups or individuals. Greater discretion, cooperation and creativity are possible in these new relationships and innovations are more likely to occur within organisations and between organisations (Davenport and Leitch, 2005).

Focused on technology adoption where the technological device or process itself may be a boundary object, Fox (2011: 73) states that ‘it could be considered a key role of an innovator to identify boundary objects, and to actively engage with users during development of technologies to ensure such objects can be generated, established and sustained’. A key role of specialist cardiac nurses was their work in identifying NSTEMI or possible NSTEMI patients and drawing them into a more recognisable pathway of care, thereby providing the crucial space where – as boundary objects –concerns and questions could then be brought (Fox 2011).

Cooperation around boundary objects is facilitated through agreed standardised methods (Star and Griesemer 1989). Allen (2015: 13) argues that nurses often fulfil boundary spanning roles and operate in interstices of health systems, making connections, aligning a constellation of actors through which care is delivered and being the ‘obligatory passage points’ in an organisation. Furthermore, nurse roles typically work to mobilise evolving trajectories of care, proactively identifying actions necessary for progress along a path, responsively determining reactions, temporal articulation, predicting work and assigning actions. In some senses, specialist cardiac nurses might themselves be seen as instruments of standardisation whose task and work is to legitimise further focus and work on the behalf of NSTEMI patients as boundary objects.

## Discussion

Defining boundaries as ‘sociocultural differences between groups that may lead to discontinuity in action or interaction’ (Akkerman and Bakker 2010:133) emphasises the activity, action and work that is often required at different boundaries. NSTEMI patients required hospital staff from different professional groups and departments to act towards them, on them and with them and care generally seemed to be better for NSTEMI patients when they received focused and coordinated attention (see also Keshet *et al*. 2013) or, in other words, when NSTEMI patients were enacted as boundary objects. Star and colleagues’ (1989, 1999, 2010) concept of boundary objects is analytically useful here because it helps to explain and disentangle the observed differences in treatment between STEMI and NSTEMI patients in these hospitals, and helps articulate why NSTEMI patient care was so challenging to staff. Gieryn's (1983) and Abbot's (1988) concepts of boundary work are also valuable because they help to explain the constraining context within which staff responsible for coordination had to operate and from which boundary objects needed to emerge: the different professions jostling against each other and often focused on protecting autonomy and workload.

In contrast to STEMI patients, NSTEMI patients in most hospitals had uncertain boundary object status. With a definition of boundary objects that emphasises shared language and being understood by different actors in more than one setting (Star and Griesemer 1989), the complexity of diagnosis and additional interpretive work (Allen 2015) in the first instance made it more difficult for staff to cooperate and work together around and for NSTEMI patients. In one hospital there were additional diagnostic hurdles over different subtypes of NSTEMI patients (type 1, type 2) that made it difficult for staff to agree and progress care for NSTEMI patients, and in another hospital inadequate categorisation meant that cardiac wards were overwhelmed with inappropriate admissions. Following Chreim and colleagues’ (2013) contention that a key leadership task in hospital settings is to open and close boundaries, some hospitals had closed off a whole category of patients and formally agreed not to give specialist cardiac care to patients over the age of 85. This has implications for equity and access to care; key indicators of care quality. Categorical and diagnostic boundary work was crucial to help sort through the patient identities and in the elevation of some patients to boundary object status and the marginalisation or silencing of others (Bowker and Star 1999, Lamont and Molnar 2002) that also occurs when a boundary object is being created.

Although boundary objects must be continually worked on (Thomas *et al*. 2007), discharge lounges and dual‐condition wards were marginal or liminal locations where NSTEMI patients could get stranded and where the clinical responsibility for their care often became ambiguous. The use of remote telemetry further undermined the potential status of NSTEMI patients as boundary objects. With remote telemetry, there was increasing fragmentation and ambiguity for staff around who had responsibility for monitoring different aspects of NSTEMI patients’ bodies and when action was required it was for an immediate urgent response. This limited the scope for negotiation and cooperation and instead revealed and exacerbated occupational, vertical and horizontal power relations (Thomas *et al*. 2007). At crucial ‘pinch points’ in the NSTEMI care pathway, such as in the catheter laboratory where angiography was carried out, the ‘silo mentality’ and lack of shared agreement and boundary object status for cardiac patients were clearly visible. In this case, because hospital spaces partly aligned with professional jurisdictions and responsibilities (Prior 1988), location mattered in the maintenance of boundary object status.

Hospital environments, characterised by constrained relations and hierarchies, fitted well with Clegg's (1989) description of the episodic circuits of power. Into this environment, NSTEMI patients with their challenging diagnostic process and variable treatment needs typically struggled to reach boundary object status. Where NSTEMI patients failed as boundary objects, the more common constraints of episodic power such as traditional doctor–nurse hierarchies (see also Powell and Davies 2010), ward / condition / disciplinary loyalties remained dominant (Abbott 1988, Gieryn 1983, Nancarrow and Borthwick 2005) and inhibited communities of practice coming together effectively for NSTEMI patients in the way that they did for STEMI patients. With support of cardiac specialists, empowered staff, and facilitatory circuits of power, NSTEMI patients could sometimes emerge as successful boundary objects and obtain the benefits that resulted from that attention.

## Conclusions

The boundary problems encountered by NSTEMI patients are evidence of a constant and necessary negotiation that is required by staff between tightly coupled elements of a complex social system. Boundary work is an everyday production and reproduction of hospital organisation, a continual accomplishment. While boundary work constantly occurred in all hospitals, in some hospitals NSTEMI patients were elevated to successful boundary object status, enabled by innovative cardiac specialist nurse roles. Traditional hierarchical power relations and parallel marginalisation help to explain why NSTEMI patients only occasionally reached boundary object status and our findings raise moral and social questions about systematic inequalities certain patient groups experienced within institutions and what is believed to be appropriate care for different age groups. Modern health care is fragmented into specialisms according to parts of the body that need attention (cardiology, renal, respiratory), the age of the patient (gerontology, paediatrics), the urgency of treatment (acute, chronic) and the professional training and roles (nurse, doctor) required to respond. To address this specialisation and fragmentation there is a greater need for interdisciplinary and cross sectional work, which brings the issue of boundaries and patients as boundary objects into particular focus (Allen 2015, Akkerman and Bakker 2010, Chreim *et al*. 2013, Nancarrow and Borthwick 2005, Powell and Davies 2012). Fox (2011) suggests identifying beliefs, values and significances of a community of practice could be useful before attempts to innovate or make improvements (2011). The lens of boundary work and boundary object theory could inform reviews of current organisational practice around patient groups that pose interdisciplinary, diagnostic and management challenges.
